# Validation of GLIM criteria for diagnosis of malnutrition in stroke survivors

**DOI:** 10.3389/fnut.2025.1644840

**Published:** 2025-09-26

**Authors:** Norah Alshammari, Alanoud Aladel, Mahmoud Desoky, Danyah Althuneyyan, Hissah Altimyat, Fatimah Alsoqeah, Mahmoud M. A. Abulmeaty

**Affiliations:** ^1^Community Health Sciences Department, College of Applied Medical Sciences, King Saud University, Riyadh, Saudi Arabia; ^2^Internal Medicine Department, Sultan Bin Abdulaziz Humanitarian City, Riyadh, Saudi Arabia; ^3^Clinical Nutrition Department, Sultan Bin Abdulaziz Humanitarian City, Riyadh, Saudi Arabia

**Keywords:** GLIM, malnutrition, stroke survivors, validity, nutrition assessment

## Abstract

**Background/objectives:**

Nutritional assessment is essential for delivering optimal care and achieving the best possible outcomes for stroke survivors. The Global Leadership Initiative on Malnutrition (GLIM) proposed new consensus criteria for diagnosing malnutrition in 2018. These criteria are anticipated to effectively predict significant outcomes related to malnutrition. This study aims to validate the GLIM criteria as a diagnostic tool for malnutrition among Saudi stroke survivors, comparing it with the subjective global assessment (SGA).

**Methods:**

A cross-sectional study was conducted involving 104 adult stroke survivors. Nutritional risk was first evaluated using the Nutrition Risk Screening 2002 (NRS-2002) as an initial step of the GLIM criteria, followed by diagnosis using both the GLIM criteria and the SGA. The level of agreement between the tools was assessed using the kappa coefficient (*κ*) statistics. Additionally, the area under the receiver operating characteristic curve (AUC-ROC) analysis was performed to determine the sensitivity, specificity, positive predictive value, and negative predictive value of the tools, thereby evaluating their accuracy.

**Results:**

A total of 104 stroke survivors were evaluated, with an average age of 61.0 years (interquartile range (IQR): 15 years), and 73.1% were men. According to the GLIM criteria, malnutrition was identified in 47.1% of the survivors, while the SGA indicated malnutrition in 51.9%. The GLIM criteria demonstrated acceptable performance, with an AUC of 0.819 (95% CI, 0.734–0.905), a sensitivity of 78.2%, and a specificity of 85.7%. The level of agreement between the two tools was substantial (*κ* = 0.635).

**Conclusion:**

The GLIM criteria for diagnosing malnutrition showed good criterion validity and appear to be a reliable approach for assessing nutritional status among stroke survivors.

## 1 Introduction

Stroke is a significant cause of death and disability worldwide, and its impact is growing over time (1). According to estimates, approximately 13.7 million new cases of stroke occur worldwide each year (2). The Kingdom of Saudi Arabia (KSA) has a significant burden of stroke, with the incidence rate expected to double by 2030 (3). Stroke is the second most common cause of death in KSA (4). One of the major complications after stroke is dysphagia, affecting over 50% of stroke survivors (5). Furthermore, stroke survivors are at a high risk of developing malnutrition and nutritional issues due to various factors, mainly associated with neurological complications (6).

Malnutrition is prevalent among stroke survivors and has been associated with a higher risk of complications, prolonged hospital stays, reduced functional improvement during rehabilitation, and elevated mortality rates (7). Malnutrition affects 6 to 62% of stroke survivors (7). Furthermore, stroke survivors face a high risk of malnutrition, which significantly impedes recovery and long-term health (7, 8). Therefore, accurate and early identification is critical for timely nutritional intervention and improved outcomes (9).

Nutritional assessment is the first and most crucial step in nutrient management (10). In recent decades, several nutritional screening tools have been developed and put into practice. The European Society for Clinical Nutrition and Metabolism (ESPEN) endorses the Nutrition Risk Screening 2002 (NRS-2002) as the standard tool for screening, recognizing it as a reliable method for evaluating the risk of malnutrition (11–13). In addition, the subjective global assessment (SGA) is a clinical tool designed to evaluate nutritional status by integrating anthropometric, biochemical, and other indicators (14). It is a widely recognized tool for identifying disease-related malnutrition due to its safety, simplicity, and cost-effectiveness (14). While it has some limitations, the SGA is regarded as the gold standard for validating other nutritional assessment methods (15).

Recently, a core leadership committee composed of representatives from major global clinical nutrition societies, including the American Society for Parenteral and Enteral Nutrition (ASPEN), ESPEN, the Federación Latinoamericana de Terapia Nutricional, Nutrición Clínica y Metabolismo (FELANPE), and the Parenteral and Enteral Nutrition Society of Asia (PENSA), was formed to standardize adult malnutrition criteria and create a globally accepted tool for diagnosing malnutrition across various clinical settings (16). The Global Leadership Initiative on Malnutrition (GLIM) was established in 2019 to address the increasing global burden of malnutrition, which includes undernutrition, overweight, and obesity. The GLIM approach involves two steps: the first step is risk screening to identify at-risk patients using any validated screening tool, such as the Nutritional Risk Screening 2002 (NRS2002), and the second step is the assessment (diagnosis) of malnutrition. The GLIM outlines specific phenotypic criteria (e.g., weight loss, low BMI, reduced muscle mass) and etiological criteria (e.g., reduced food intake, inflammation) that must be met for a malnutrition diagnosis. GLIM represents a consensus that requires validation for clinical application (16). Additional evidence is necessary to confirm the validity of the GLIM criteria. To explore this, we hypothesized that GLIM is a reliable tool for diagnosing malnutrition among Saudi stroke survivors when compared to the Subjective Global Assessment (SGA). Therefore, the primary objective of this study was to validate the use of GLIM criteria as a diagnostic tool for malnutrition in stroke survivors in comparison to SGA (16).

## 2 Materials and methods

### 2.1 Sample selection and study design

A cross-sectional study was conducted between January and April 2024 at the stroke rehabilitation facilities of Prince Sultan bin Abdulaziz Humanitarian City (SBAHC) in Riyadh, Saudi Arabia. The study population included males and females aged ≥18 years or older with a history of stroke who were admitted to the hospital during this period. Exclusion criteria encompassed patients aged ≤18 years, those with hemiparesis or contracture deformities that could affect anthropometric assessments, pregnant or lactating individuals, and patients with incomplete reports. The study received approval from the SBAHC Ethical Committee in Riyadh, KSA (IRB No. 86–2022), and informed consent was obtained from all participants. MedCalc Statistical Software was utilized to determine the sample size based on the area under the curve (AUC), using SGA as the gold standard to assess the validity of GLIM. A total of 94 stroke survivors were required for the study. This calculation was based on an alpha level of 0.05, a beta level of 0.10 (90% power), and a minimum expected AUC of 0.70 (indicating a moderately accurate test: ≥ 0.7 and ≤ 0.9), with a null hypothesis value of 0.5, referencing a previous study that applied GLIM to evaluate nutritional status in ICU patients (17). Anticipating a 10% dropout rate, we aimed for a total sample size of 104 participants.

### 2.2 Sociodemographic and clinical history

The survey collected the following sociodemographic and clinical information: age, gender, marital status, duration of stroke, and type of stroke. Additionally, participants were asked whether they always have comorbidities and complications (Yes or No) such as hypertension, diabetes mellitus, dyslipidaemia, thyroid disease, cardiovascular disease, nephropathy, and others.

### 2.3 Nutrition screening and assessment

Participants were assessed using the NRS 2002 and SGA. Some questions overlapped between the two tools, including body mass index (BMI), weight loss, and decreased food intake. To minimize the burden on participants, these questions were only asked once. A participant was considered at risk of malnutrition if they scored three or higher on the NRS 2002 (18). In SGA classified with categories B (moderately malnourished) or C (severely malnourished) (14). This study utilized the malnutrition diagnostic criteria proposed by the GLIM, which includes five criteria: non-volitional weight loss, low body mass index, reduced muscle mass, decreased food intake or assimilation, and disease burden/inflammation (16). According to GLIM, a participant is diagnosed with malnutrition if they meet at least one phenotypic criterion and one etiological criterion. Since the skeletal muscle index was not measured in this study, alternative indicators such as MUAC and CC were used. Cut-off points for low muscle mass were established by the Asian Working Group for Sarcopenia in 2019 and supported by other studies (19, 20).

### 2.4 Anthropometric measurements

#### 2.4.1 Body mass index (BMI)

Body weight was measured by a trained clinical dietitian using an electronic scale with an accuracy of 0.1 kg, while body height was recorded with a portable stadiometer to the nearest 0.1 cm. The participants’ BMI was determined using Quetelet’s index, calculated as BMI = body weight (kg) / height (m^2^). A low BMI was defined as less than 18.5 kg/m^2^ for individuals under 70 years old and less than 20 kg/m^2^ for those aged 70 years and older (16).

#### 2.4.2 Weight loss (WL% %)

Participants were initially asked about their usual weight. Their current weight was obtained using a weighing scale compared to their usual weight. If a participant experienced weight loss (non-volitional), the percentage of weight loss (WL%) was calculated using the following formula: Percent weight loss = (Usual weight - Current weight) / Usual weight x 100. Unintentional weight loss was defined as a decrease of more than 5% within the past 6 months or more than 10% beyond 6 months (16).

#### 2.4.3 Waist circumference (WC)

The WC measurements followed the guidelines set by the World Health Organization (WHO) (21). Using a measuring tape, WC was measured at the midpoint between the lowest rib and the iliac crest in a horizontal plane.

#### 2.4.4 Mid-upper arm circumference (MUAC) and calf circumference (CC)

The research team assessed decreased muscle mass by measuring calf circumference (CC) and mid-upper arm circumference (MUAC) during physical examinations. MUAC was measured on the non-paralyzed arm at the midpoint of the mid-upper arm, ensuring the elbow was fully extended. Results were recorded to the nearest 0.1 cm. To identify the midpoint, the right arm was bent at a 90° angle at the elbow, which is located halfway between the olecranon process of the ulna and the acromion process of the scapula. Similarly, CC was measured while the subject was seated or lying down, with the non-paretic knee flexed at a 90-degree angle. According to the Asia Working Group for Sarcopenia, decreased calf circumference is defined as less than 34 cm for males and less than 33 cm for females (19).

#### 2.4.5 Triceps skinfold (TSF)

Thickness was measured at the midpoint of the posterior line between the olecranon and the tip of the acromion. The research team performed the measurements using digital skinfold fat calipers, recording them to the nearest 0.5 mm.

#### 2.4.6 The mid-arm muscle circumference (MAMC)

MAMC was calculated using the standard formula: MAMC (centimeters) = MUAC (centimeters) − *π* × (TSF thickness [millimeters] ÷ 10) (22).

### 2.5 Biochemical data

Laboratory measurements, including total cholesterol, low-density lipoprotein (LDL), high-density lipoprotein (HDL), hemoglobin (HGB), albumin, neutrophil-to-lymphocyte ratio (NLR), total protein, and creatinine, were obtained from patients’ electronic medical records, with data collected within 3 months of the assessment date.

### 2.6 Statistical analysis

Statistics were calculated using the Statistical Package for Social Sciences (SPSS) software, version 22. Normality of the parameters was tested by the Shapiro–Wilk test. Continuous variables were reported as mean ± standard deviation (SD) or median and interquartile range (IQR), depending on the normality of the data. Categorical variables were shown as counts (n) and percentages (%). The Chi-square test or Fisher’s exact test was used for analyzing these categorical variables. For continuous data, the Student’s t-test was applied for normally distributed variables. At the same time, the Mann–Whitney U test was used to compare patients with and without malnutrition, as the variables were non-normally distributed. The agreement between the tools was evaluated using the kappa coefficient (*κ*), with a required validity threshold for GLIM set at > 0.80 (23). The area under the receiver operating characteristic curve (AUC-ROC), along with a 95% confidence interval (CI), sensitivity (Se), specificity (Sp), and positive and negative predictive values (PPV and PNV), were calculated to evaluate the concurrent validity of the GLIM criteria, using SGA as the reference method. A *p*-value < 0.05 was considered statistically significant.

## 3 Results

### 3.1 Characteristics and nutritional status of stroke survivors

**Figure 1:**
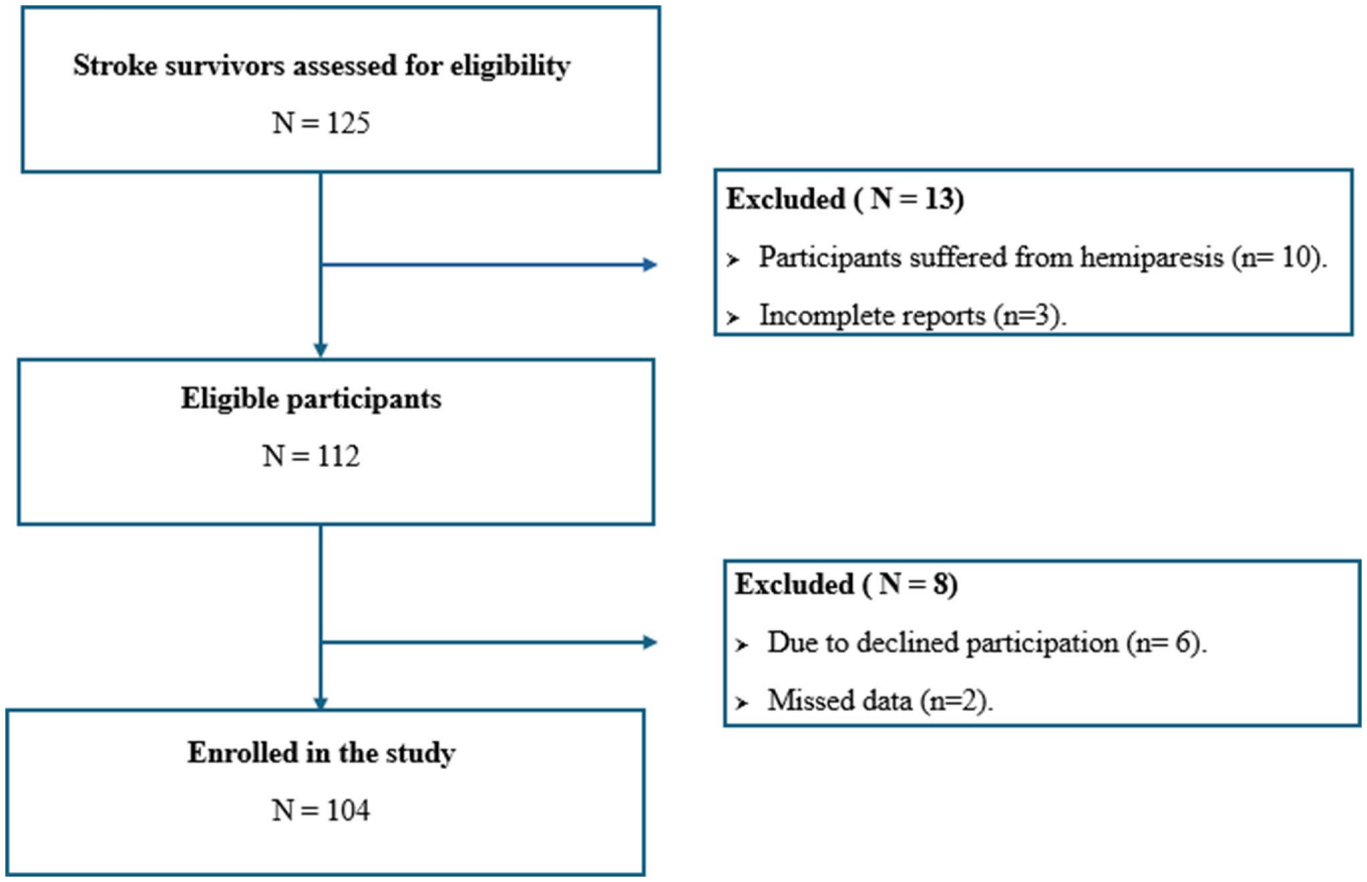
Flowchart of study participants.

Table 1 shows the characteristics of the stroke survivors enrolled in the study (Figure 1). A total of 104 stroke survivors, 76 males 73.1% and 28 females 26.9%, ranging in age from 21 to 84 years [median 61.0 years, interquartile range (IQR): 15 years]. Approximately 94.2% of survivors were married. The median duration of stroke was 24 months, IQR: 27 months. According to stroke type, the majority of survivors have ischemic strokes 65.4%, while 34.6% have hemorrhagic strokes. Hypertension, diabetes mellitus, and dyslipidemia were considered the main comorbidities, in which they were present in 81.7, 60.6, and 51.0% of survivors, respectively. Survivors suffer macrovascular complications 24.0%, and microvascular complications 5.8%. According to nutritional status, using NRS-2002 to identify individuals at risk of malnutrition, it was found that 55.8% of survivors were at risk of malnutrition. According to GLIM criteria, 47.1% of survivors were diagnosed with malnutrition, whereas 51.9% were diagnosed with malnutrition due to SGA.

**Table 1:** Characteristics and nutrition status of stroke survivors.

Variable	Total (104)
Age*	61.0 (15)
Gender**	
Male	76 (73.1%)
Female	28 (26.9%)
Marital status**	
Single	4 (3.8%)
Married	98 (94.2%)
Divorced	2 (1.9%)
Duration of Stroke* (months)	24.0 (27)
Stroke type**	
Ischemic stroke	68 (65.4%)
Hemorrhagic stroke	36 (34.6%)
Comorbidities**	
HTN	85 (81.7%)
DM	63 (60.6%)
DLP	53 (51.0%)
Thyroid disease	12 (11.5%)
Other	8 (7.7%)
Macrovascular complication**	
Cardiovascular disease	25 (24.0%)
Microvascular complication**	
Nephropathy	6 (5.8%)
Nutritional status**	
GLIM	
Well-nourished	55 (52.9%)
Malnourished	49 (47.1%)
SGA	
Well-nourished	50 (48.1%)
Malnourished	54 (51.9%)
NRS-2002	
Not At risk of malnutrition	46 (44.2%)
At risk of malnutrition	58 (55.8%)

HTN, Hypertension; DM, Diabetes Mellitus; DLP, Dyslipidemia; GLIM, Global Leadership Initiative on Malnutrition; SGA, Subjective global assessment; NRS-2002, Nutrition Risk Screening 2002. * Data were presented as median & (IQR), ** data were presented as number and percentage *n* (%).

### 3.2 Anthropometric characteristics of stroke survivors according to GLIM criteria and SGA

Table 2 displays anthropometric status categorized by nutritional status using GLIM and SGA criteria, respectively. The results revealed a significant difference between well-nourished and malnourished survivors in terms of weight, BMI, MUAC, CC, and MAMC according to both GLIM and SGA criteria.

**Table 2:** Anthropometric characteristics of stroke survivors according to GLIM and SGA.

Screening tools						
Variable	GLIM Well-nourished (*n* = 55)	GLIM Malnourished (*n* = 49)	GLIM *p-*value	SGA Well-nourished (*n* = 50)	SGA Malnourished (*n* = 54)	SGA *p-*value
Anthropometric*						
Weight	75.24 ± 15.62	64.80 ± 13.78	0.001^a^	74.60 ± 14.46	66.36 ± 15.73	0.007^a^
Height	165.94 ± 8.26	165.33 ± 8.59	0.713^a^	166.05 ± 6.90	165.28 ± 9.60	0.641^a^
BMI	27.08 ± 4.75	23.66 ± 4.69	0.000^a^	26.86 ± 4.52	24.18 ± 5.12	0.006^a^
WC	97.15 ± 15.15	95.84 ± 14.75	0.657^a^	97.14 ± 16.03	95.96 ± 13.90	0.689^a^
MUAC	31.78 ± 3.37	28.12 ± 3.90	0.000^a^	31.96 ± 3.13	28.30 ± 4.04	0.000^a^
CC	34.27 ± 2.46	31.69 ± 3.26	0.000^a^	34.36 ± 2.35	31.85 ± 3.30	0.000^a^
TSF	21.91 ± 6.01	23.54 ± 6.15	0.176^a^	22.21 ± 6.18	23.12 ± 6.05	0.452^a^
MAMC	23.86 ± 2.70	21.71 ± 2.61	0.000^a^	24.19 ± 2.54	21.61 ± 2.58	0.000^a^

WC, Waist Circumference; MUAC, Mid-upper arm circumference; CC, Calf Circumference; TSF, Triceps skinfold thickness; MAMC, Mid arm muscle circumference. * Data were presented as mean ± standard deviation (SD), *p*-value < 0.05 is considered statistically significant.

a Student *t*-test.

### 3.3 Biochemical characteristics of stroke survivors according to GLIM criteria and SGA

Table 3 shows the biochemical characteristics of stroke survivors according to GLIM and SGA criteria. The results demonstrated an insignificant difference between well-nourished and malnourished according to GLIM, with a *p*-value > 0.05, while according to the SGA tool, there was a significant difference in total cholesterol between malnourished and well-nourished groups with a *p*-value < 0.05.

**Table 3:** Biochemical characteristics of stroke survivors according to GLIM and SGA.

Screening tools						
Variable	GLIM Well-nourished (*n* = 55)	GLIM Malnourished (*n* = 49)	GLIM *p-*value	SGA Well-nourished (*n* = 50)	SGA Malnourished (*n* = 54)	SGA *p-*value
Total cholesterol (mmol/L)	3.75 ± 1.32	3.33 ± 0.78	0.148	3.84 ± 1.38	3.28 ± 0.71	0.049
LDL (mmol/L)	2.10 ± 0.88	1.78 ± 0.80	0.144	2.12 ± 0.98	1.79 ± 0.69	0.134
HDL (mmol/L)	0.98 ± 0.29	0.97 ± 0.32	0.890	1.04 ± 0.29	0.92 ± 0.30	0.119
NLR	2.39 ± 3.13	2.70 ± 2.09	0.590	2.48 ± 3.27	2.59 ± 1.99	0.858
HGB (g/dL)	12.59 ± 2.10	12.76 ± 1.92	0.678	12.81 ± 2.03	12.54 ± 2.00	0.527
Creatinine (umol/L)	72.44 ± 47.18	66.38 ± 20.61	0.449	75.37 ± 48.60	63.93 ± 19.25	0.151
Albumin (g/L)	36.16 ± 3.80	35.06 ± 4.90	0.274	36.19 ± 3.95	35.18 ± 4.67	0.313
Total protein (g/L)	78.32 ± 16.80	74.13 ± 11.15	0.142	76.83 ± 13.80	75.90 ± 15.24	0.764

LDL, Low-density lipoprotein; HDL, High-density lipoprotein; NLR, Neutrophil-to-Lymphocyte Ratio; HGB, Hemoglobin. Data were presented as mean ± standard deviation (SD). *p*-value < 0.05 is considered statistically significant.

Student *t*-test.

### 3.4 Comorbidities and complications of stroke survivors according to GLIM criteria and SGA

Additionally, according to the GLIM criteria, there was a significant difference in dyslipidemia and thyroid disease between malnourished and well-nourished groups, with a *p*-value <0.05, while according to the SGA tool, there was a significant difference in dyslipidemia and hypertension between malnourished and well-nourished groups, with a *p-*value < 0.05 (see Table 4).

**Table 4:** Comorbidities and complications of stroke survivors according to GLIM and SGA.

Screening tools						
Variable	GLIM Well-nourished (*n* = 55)	GLIM Malnourished (*n* = 49)	GLIM *p-*value	SGA Well-nourished (*n* = 50)	SGA Malnourished (*n* = 54)	SGA *p-*value
Comorbidities						
HTN	43 (78.2%)	42 (85.7%)	0.321^a^	37 (74.7%)	48 (88.9%)	0.050^a^
DM	32 (58.2%)	31 (63.3%)	0.596^a^	28 (56.0%)	35 (64.8%)	0.358^a^
DLP	33 (60.0%)	20 (40.8%)	0.051^a^	33 (66.0%)	20 (37.0%)	0.003^a^
Thyroid Disease	10 (18.2%)	2 (4.1%)	0.025^a^	9 (18.0%)	3 (5.6%)	0.416^a^
Other	4 (7.3%)	4 (8.2%)	1.000^b^	2 (4.0%)	6 (11.1%)	0.273^b^
Macrovascular complications						
CVD	12 (21.8%)	13 (26.5%)	0.575^a^	12 (24.0%)	13 (24.1%)	0.993^a^
Microvascular complication						
Nephropathy	3 (5.5%)	3 (6.1%)	1.000^b^	3 (6.0%)	3 (5.6%)	1.000^b^

HTN, Hypertension; DM, Diabetes Mellitus; DLP, Dyslipidemia; CVD, Cardiovascular Diseases * Data were presented as number and percentage *n* (%). *p*-value < 0.05 is considered statistically significant.

a Chi-square test.

b Fisher test.

### 3.5 Concurrent validity of GLIM criteria

**Figure 2:**
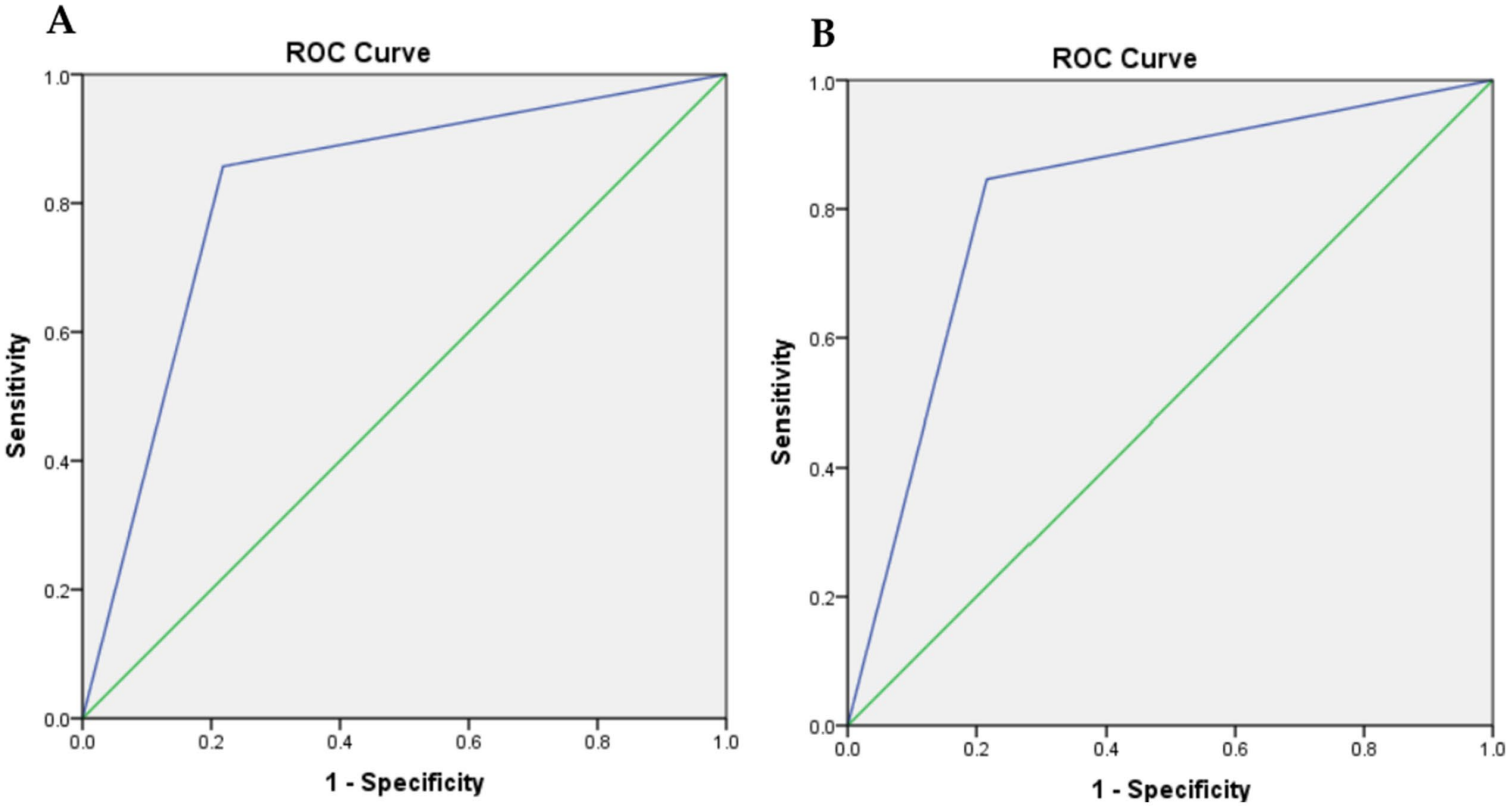
Receiver-operating characteristic (ROC) curve plot of the true positive rate (sensitivity) against the false positive rate (1-specificity) at GLIM criteria compared with SGA. **(A)** for all cases, and **(B)** for males only.

The GLIM criteria were validated through kappa and ROC analysis, using SGA as the reference (Table 5). The results indicated a substantial agreement between the GLIM criteria and SGA (*κ* = 0.635, *p* = 0.000). ROC analysis revealed that the GLIM criteria demonstrated good sensitivity (78.2%) and excellent specificity (85.7%) in comparison to SGA. The area under the curve (AUC) indicated a strong ability of the GLIM criteria to diagnose malnutrition (AUC = 0.819; 95% CI, 0.734–0.905). Based on the predominance of males in this study, we did a validity assessment in males only and found similar values to the results of the total sample (Table 6, Figure 2).

**Table 5:** Concurrent validity of GLIM criteria using SGA as reference.

Statistical parameters of concurrent validity		
Kappa (κ)	0.635	*p*-value (<0.001)
AUC (CI 95%)	0.819 (0.734–0.905)	*p*-value (<0.001)
Sensitivity	78.2%	
Specificity	85.7%	
Predictive Positive Value	86.0%	
Predictive Negative Value	77.8%	

AUC, Area Under Curve; C.I., confidence interval. *p*-value < 0.05 is considered statistically significant.

**Table 6:** Concurrent validity of GLIM criteria using SGA as a reference for male cases only.

Statistical parameters of concurrent validity		
Kappa (*κ*)	0.631	*p*-value (<0.0001)
AUC (CI 95%)	0.815 (0.713–0.917)	*p*-value (<0.0001)
Sensitivity	80.5%	
Specificity	82.9%	
Predictive positive value	84.6%	
Predictive negative value	78.4%	

AUC, Area Under Curve; C.I., confidence interval. *p*-value < 0.05 is considered statistically significant.

### 3.6 Prevalence of GLIM criteria among stroke survivors

According to phenotypic criteria, 71.2% of survivors did not experience any volitional weight loss, while low BMI was observed in 6.7%, and reduced muscle mass was identified in 63.5%. Regarding the etiological criteria, low food intake and inflammation were present in 59.6 and 32.7% of survivors, respectively. Notably, the percentages of survivors with weight loss, low BMI, and reduced muscle mass were significantly higher (*p*-value < 0.05) in the malnourished group compared to the well-nourished group (91.8% vs. 52.7, 14.3% vs. 0.0, and 87.8% vs. 41.8%, respectively), as shown in Table 6. Additionally, for the etiological criteria, the percentages of survivors with low food intake and disease/inflammation were significantly greater in the malnourished group compared to the well-nourished group (89.8% vs. 32.7 and 49.0% vs. 18.2%, respectively), as indicated in Table 7.

**Table 7:** Prevalence of phenotypic and etiologic components of GLIM criteria among stroke survivors.

GLIM				
Variable	Total (*n* = 104)	Well-nourished (*n* = 55)	Malnourished (n = 49)	*p-*value
Phenotypic criteria				
Weight loss	74 (71.2%)	29 (52.7%)	45 (91.8%)	0.000^a^
Low BMI	7 (6.7%)	0 (0.0%)	7 (14.3%)	0.004^b^
Reduce Muscle Mass	66 (63.5%)	23 (41.8%)	43 (87.8%)	0.000^a^
Etiologic criteria				
Low Food Intake	62 (59.6%)	18 (32.7%)	44 (89.8%)	0.000^a^
Disease/Inflammation	34 (32.7%)	10 (18.2%)	24 (49.0%)	0.001^a^

BMI, Body Mass Index. Data for categorical variables were expressed as a number and percentage n (%). *p*-value < 0.05 is considered statistically significant.

a Chi-square test.

b Fisher test.

## 4 Discussion

The present study aimed to validate the use of GLIM criteria as a diagnostic tool for malnutrition in stroke survivors, in comparison to the SGA. Our findings indicate that the GLIM criteria demonstrate acceptable performance in diagnosing malnutrition, with an area under the receiver operating characteristic curve (AUC) of 0.819. The sensitivity was found to be 78.2%, and the specificity was excellent at 85.7% when compared to the SGA. Furthermore, both GLIM and SGA showed significant correlations with various nutritional parameters, including weight, BMI, MUAC, CC, and MAMC.

The SGA has been widely used worldwide and validated across various clinical settings with different populations. The GLIM criteria group considers it a “semi-gold” standard (16). In addition, several studies have validated GLIM criteria compared to SGA among different populations (24–26).

Our study revealed a significant prevalence of malnutrition among stroke survivors, with rates of 47.1% according to the GLIM criteria, comparable to Brito et al. (26) at 41.6%. Concordance between GLIM and SGA was strong, with 51.9% malnutrition identified by SGA, consistent with Balci et al. (25) (*κ* = 0.804). However, our study’s findings differ from Allard et al. (27), who reported lower GLIM sensitivity (61.3%). On the other hand, our findings indicated greater effectiveness in identifying malnourishment among stroke survivors, underscoring the significance of GLIM in advancing prompt nutritional therapies and enhancing recuperation results (16).

When comparing the well-nourished and malnourished stroke survivors by GLIM and SGA, there was a significant difference in weight, BMI, MUAC, CC, and MAMC (*p* < 0.05). For instance, the weight of a well-nourished survivor using GLIM was 75.24, while that of the malnourished survivor was 64.80, *p* = 0.001. This means that underweight stroke survivors would have weighed significantly less compared to those who were well-nourished. These findings are further supported by the BMI readings: well-nourished subjects had a BMI of 27.08 kg/m^2^, while those malnourished had a BMI of 23.66 kg/m^2^ (*p* = 0.000). This agrees with Speranza et al. (28), who, after conducting their research, reported that lower values of both BMI and CC were found to be significantly associated with higher nutritional risk, thus underlining once again the importance of this measure in the identification of malnutrition, as well as placing emphasis on the nutritional status concerning recovery and rehabilitation outcomes post-stroke. The consistent findings between the GLIM and SGA criteria further underscore their clinical utility for the diagnosis of malnutrition, with both tools indicating significant differences in important anthropometric measures of nutritional status. These findings indicate that a gross compromise in muscle mass and general physical health, characterized by compromised anthropometric measures such as MUAC and CC, is a key determinant of recovery following stroke.

Evaluation of biochemical aspects of stroke survivors based on GLIM and SGA criteria provides interesting insights into the diagnosis of malnutrition. The minor differences observed in biochemical markers, especially in Table 3, align with the results reported by Fiorindi et al. (29), stating that simple, routine nutritional indicators are sometimes insufficient to determine malnutrition among patients. As shown in Table 2, most of the biochemical markers recorded were similar between well-nourished and malnourished groups (*p*-values > 0.05), with a slight trend in total cholesterol, the values for which were lower among malnourished (3.33 ± 0.78 mmol/L) compared to well-nourished (3.75 ± 1.32 mmol/L). This aligns with other studies that have demonstrated that low cholesterol levels can indicate malnutrition in stroke patients, potentially resulting from insufficient nutrient intake or changes in cholesterol metabolism. In contrast, Table 3 shows a significant difference in total cholesterol levels based on SGA (*p* = 0.049), which is consistent with the findings of Galindo Martín et al. (30), who reported a correlation between malnutrition identified by GLIM criteria and adverse clinical outcomes. Their findings indicate that cholesterol levels may play a crucial role in evaluating nutritional status and predicting complications across different patient groups. Consequently, albumin and hemoglobin, which are biochemical markers of malnutrition, might not effectively distinguish between stroke patients with malnutrition and those who are well-nourished. Combining GLIM and other clinical criteria with comprehensive assessments could enhance nutritional interventions.

According to the GLIM criteria, there were no significant differences in the incidence of diabetes mellitus (DM), hypertension (HTN), and dyslipidemia (DLP) between malnourished and non-malnourished patients, with *p* > 0.05. This suggests that the GLIM criteria may not effectively capture the complex relationships between malnutrition and specific comorbidities in this patient population. However, the SGA tool showed a *p*-value of 0.050 in HTN and 0.003 in DLP between the malnourished and well-nourished groups. These findings show that SGA is more useful in determining the relationship between nutrition and these diseases. The strong association with DLP corresponds with a previous study, which notes that malnutrition may worsen disorders in lipid metabolism and contribute to worse cardiovascular outcomes (7). Thereby, despite the promising findings regarding the application of GLIM for the diagnosis of malnutrition, SGA may offer a better understanding of the connection between malnutrition and comorbidities, including DLP and HTN, which supports the necessity for the use of multiple screening tools in clinical practice to enhance the approach to the patient.

The agreement level between the GLIM criteria and the SGA was *κ* = 0.635, indicating substantial concordance. These findings are consistent with earlier studies across different populations that have validated the GLIM criteria against the SGA. For instance, a retrospective study by Zhang et al. (31) involving 3,777 cancer patients reported an agreement of *κ* = 0.54 and a low sensitivity of 70%. Similarly, a retrospective cohort study of 784 hospitalized patients found that while the GLIM criteria demonstrated good specificity at 89.8%, their sensitivity was lower at 61.3% compared to the SGA (27).

Few studies have examined the prevalence of malnutrition in stroke patients using GLIM criteria. A cross-sectional study involving 304 stroke patients in the rehabilitation phase reported that 25.3% were diagnosed with malnutrition, with 67.5% categorized as severely malnourished (32). Additionally, a retrospective cohort study of 122 stroke patients revealed a higher prevalence of 64.8% (33). In contrast, our study found that 47.1% were diagnosed with malnutrition. The elevated percentage of malnourished patients in the retrospective cohort study may be attributed to the characteristics of the enrolled population, which primarily consisted of older adults (33). Furthermore, a cross-sectional study of 115 acute stroke patients indicated that 28.7% were diagnosed as malnourished (34).

It is well documented that BMI is the main significant factor influencing the risk, outcomes, and rehabilitation of stroke patients. The present study found that about 14.3% of malnourished survivors were underweight. The findings are consistent with a retrospective cohort study that observed the BMI of the majority of malnourished patients with strokes underweight range. Another study found that 22% of the patients had a low BMI (35). These findings highlight the significant proportion of stroke patients who are at risk of malnutrition due to low BMI. Additionally, another study found that 28.7% of the patients had malnutrition (34).

A key indicator of malnutrition is non-volitional weight loss, a criterion strongly highlighted by the GLIM consensus (16). In the present study, non-volitional weight loss was the predominant phenotypic criterion observed in 91.8% of malnourished survivors. This finding is consistent with a previously mentioned retrospective cohort study, which found that weight loss was the most common phenotypic criterion, affecting 83.9% of patients with stroke (33).

Another important phenotypic criterion in the GLIM evaluation of malnutrition is reduced muscle mass. In our study, reduced muscle mass was identified in 41.8% of the well-nourished group, while this percentage increased to 87.8% among malnourished individuals. This aligns with findings from a single-center cohort study of 189 patients with acute stroke, which reported low muscle mass in 46% of males and 56% of females (36). Therefore, early evaluation of muscle mass in post-stroke patients may be essential.

Regarding etiological criteria, reduced food intake was identified as the most common factor among malnourished survivors, occurring in 89.8% of cases, while disease or inflammation was present in 49% of these individuals. Inflammation was assessed using the neutrophil-to-lymphocyte ratio (NLR) in instances where albumin levels were not available. NLR is a widely recognized and easily accessible inflammatory marker. However, it is important to emphasize that low albumin is the preferred indicator of inflammation, as recommended by the GLIM group (16).

In addition to their accuracy in identifying malnutrition, clinicians must consider the strengths and limitations of each nutrition screening tool. This study is among the few that have assessed the validity of various malnutrition screening instruments, including the latest GLIM, within the rehabilitation context in Saudi Arabia. Consequently, we believe our findings provide valuable insights into the critical selection of appropriate malnutrition screening tools in stroke rehabilitation settings.

However, this study has several limitations. Since this study was conducted at a single center in Riyadh City, the generalizability of the results to the broader Saudi population is limited. Additionally, food intake was evaluated through self-reporting rather than direct dietary assessment methods. Furthermore, men and women were pooled in the analysis without sex-specific adjustments, and the proportion of female participants was relatively low compared with males, which may limit the applicability of the findings to female patients. Therefore, caution is warranted when interpreting the results in females. Future studies should apply sex-stratified analyses for more precise interpretation. Future research is needed to explore the feasibility and predictive validity of these malnutrition screening tools.

## 5 Conclusion

In conclusion, the GLIM criteria demonstrated good sensitivity and specificity, as well as acceptable criterion validity, and substantial agreement with the SGA was observed. Validation studies comparing GLIM with SGA on a larger nationwide scale are strongly recommended to further confirm these findings in stroke survivors. Additionally, it is advisable to conduct GLIM validation studies in Saudi Arabia and abroad with various patient populations to explore the potential of adopting GLIM criteria as a standardized nutritional assessment tool in different institutions and hospitals.

## Data Availability

The raw data supporting the conclusions of this article will be made available by the authors, without undue reservation.
